# Newspaper Advertising

**Published:** 1907-01

**Authors:** 


### NEWSPAPER ADVERTISING.

In a recent editorial, the *St. Louis Medical Era* justly scores some local regular members of the medical profession for permitting themselves to be "written up" in a St. Louis newspaper. The photographs and biographical sketches of these men, some of them being not only of local but national reputation, were reproduced in the *Era* as they had appeared in the lay press.

Time was when we in Georgia were not infrequently treate dto similar exhibitions of impropriety and bad taste, to phrase it mildly; but at present the quacks have the field exclusively, and but for a casual notice in the daily press, more or less justified, of some local physician, the custom has become obsolete. It is entirely right and proper that it should become obsolete for every doctor worthy of the name if he knows anything at all and whether or not he has read the code of ethics, knows that it is fundamentally and uncompromisingly wrong for him to allow himself to be the subject of a newspaper puff.

There are many who express profound surprise and indignation when they have been thus signalized and advertised, but this surprise and indignation will rarely ever pass current as genuine; and those who are wise to the ways of guile know that there was connivance or the deed would never have been possible.

Of course there are times of public stress, or epidemic, or calamity, where a doctor, for services rendered the public weal, is justly entitled to widespread recognition; but in piping times of peace, when things run smooth, he who commits that offense which of all others creates such a stench in the nostrils of those who abide by the code in spirit and in letter, is not only guilty of a great wrong, but violates the law and the dignity of his profession in a manner that excites the most profound contempt.

It is difficult for a layman to understand the situation. It seems to him absurd that a doctor is not allowed by the custom and usage of his profession to appear in the public print. He has the same privilege as a banker, a preacher, or any other reputable citizen, and why may he not avail himself of it?

It is also difficult to explain the attitude of the profession toward this question, but that the attitude is one of unalterable opposition there can be no doubt. Its origin is perhaps based upon the requirement of secrecy, discretion and trustworthiness imposed upon the profession. Publicity is in a way opposed to these qualities, and is, therefore, unbecoming and undesirable in a doctor.

The doctor is the one man to whom the burdened heart may pour out its sorrows, its tale of shame, or of crime, or weakness, with the confident trust and belief that he is worthy of absolute confidence and that he will not betray this trust, even on the rack.

Such a person a sick man pictures as going about among the people on his benificent missions quietly, steadily, unostentatiously; not in the lime-light of newspaper publicity—with his photograph and his list appended of paltry and ephemeral honors.

Then, from the professional side, it is taking an unfair advantage over his fellow practitioners. They are not permitted by code or conscience to seek this means of advertisement and self-aggrandizement. They may not retaliate in kind; they must submit in scornful silence. If the custom were abolished, and newspapers were adopted as a means of self-praise and to acquire patients, the distinction between the quack and the honest doctor would at once disappear or become so slight as to be neglible. There would be no better way to drag the dignity of the profession in the mire than for its members to resort to the newspaper or to alter one whit the inflexible sternness of the code.

The *Medical Era* is to be congratulated upon the bold stand it has taken in denouncing the offenders, regardless of their influence, rank and reputation

---

At the meeting of the Fulton County Medical Society, December 20th, after Dr. E. Bates Block's retiring address, the following officers were elected for the ensuing year: President—Dr. Claude Smith; Vice-President—Dr. W. B. Emery; Secretary—Dr. Michael Hoke; Treasurer—Dr. E. Van Goidtsnoven. The censors are: Dr. Cyrus Strickler, Dr. Ormsted and Dr. E. C. Davis.

---

The Chinese have a saying, dating back eleven centuries before our era, that "no one can be called a physician who does not cure six out of ten patients, for, if only five recover, the patient is as well off without him, as half of all the sick get well without medical aid."

---

For the enlargement and betterment of the *Oklahoma Medical News-Journal.* Beginning with the January, 1907, issue, the *Oklahoma News-Journal* will have a new editor, Y. E. Colville, B. S., M. D., of Chattanooga, Tenn. Dr. Colville has bought a half interest in the journal, and will devote his entire time to the editorial department, while Dr. Phelan will be the business manager. In this way the journal will be greatly benefited and enlarged, and will be more valuable to the profession than heretofore.

